# Supplementary material for: Quantifying Unbiased Conformational Ensembles from Biased Simulations Using ShapeGMM
Source: J Chem Theory Comput. 2024 Apr 25;20(9):3492–502. doi: 10.1021/acs.jctc.4c00223 (PMC11104435; doi:10.1021/acs.jctc.4c00223)
Supplement: Supplementary file 1 — ct4c00223_si_001.pdf [file ct4c00223_si_001.pdf]

Supporting Information For: Quantifying Unbiased Conformational Ensembles from Biased Simulations Using ShapeGMM

Subarna Sasmal,[†] Triasha Pal,[†] Glen M. Hocky,[*,†,¶] and Martin McCullagh[*,‡]

†*Department of Chemistry, New York University, New York, NY 10003*

‡*Department of Chemistry, Oklahoma State University, Stillwater, OK 74078*

¶*Simons Center for Computational Physical Chemistry, New York University, New York, NY 10003*

E-mail: hockyg@nyu.edu; martin.mccullagh@okstate.edu

S1 Choosing Training Data

When fitting a shapeGMM, we split our data into a training set and a cross validation set. The Gaussian mixture components are fit on the training data and their ability to model the cross validation set is assessed by comparing the log likelihood per frame on both sets. Overfitting will lead to a lower log likelihood on the cross validation set than on the training set. Both training and prediction routines now have built in frame weight arguments.

Training sets were chosen uniformly randomly for the original implementation of shapeGMM. For non-uniform frame weights, however, there are a variety of other methods one could consider to best choose a training set. We assessed a number of these including simple ranking, Poisson sampling, and a Metropolis Monte Carlo method using log frame weights as energies. It was found the the uniform sampling of frame weights worked as well as other methods especially when training sets are sufficiently large.

A uniform sampling of the training set performs at least as well as importance sampling of the training set for the beaded helix example. To assess this we compared shapeGMM objects fit using various training set sampling schemes. These include: a uniform sampling, a Monte Carlo sampling in which frames are replaced based on the Metropolis criteria using frame weights, and a Poisson sampling scheme in which

frames are sampled from the frame weight distribution. The Poisson sampling method differs from the other two in that frames are equally weighted in the training set but can appear multiple times depending on their relative weights. The Jensen-Shannon divergence (JSD) between distributions fit using these methods to an $\epsilon = 6$ trajectory with $\epsilon = 8$ weights and distributions fit to an $\epsilon = 8$ simulation directly (the ground-truth; GT) as a function of training set size are depicted in Fig. S1. The JSD between the GT and all fitted distributions is large (~ 0.3) for small training set sizes and tends to zero as training sets increase. This indicates that all methods are accurately reproducing the GT distribution for large enough training set. We find that the uniform sampling approach does as well or better than either importance sampling approach for all training set sizes. We note that this result will depend on the specific distribution of weights. We expect this behavior to hold for relatively uniform distributions of weights which occur in reweighting to Hamiltonians that don't deviate much from the original. It may be important, especially for small training set sizes, in cases in which the Hamiltonians are significantly different to consider choosing training sets using an importance sampling approach. We use a uniform sampling approach for all other applications in this paper.

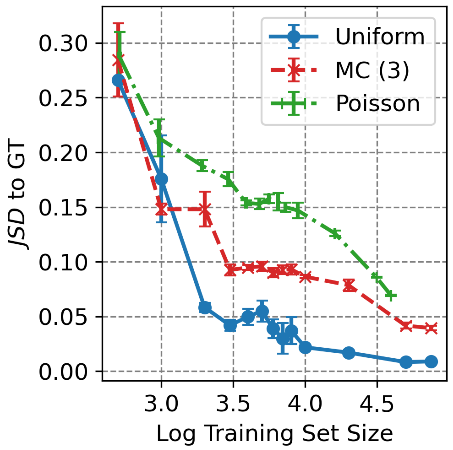

Figure S1: Accuracy of beaded helix reweighted cluster as a function of training set size. The Jensen-Shannon divergence (JSD) between shapeGMM distribution fit using reweighting to $\epsilon = 8$ and the ground-truth fit to a simulation run at $\epsilon = 8$ as a function of training set size. Three taining set selection schemes are compared: a uniform sampling of frames, a three-step Monte Carlo importance sampling method, and a Poisson sampling method.

S2 Clustering untempered metadynamics

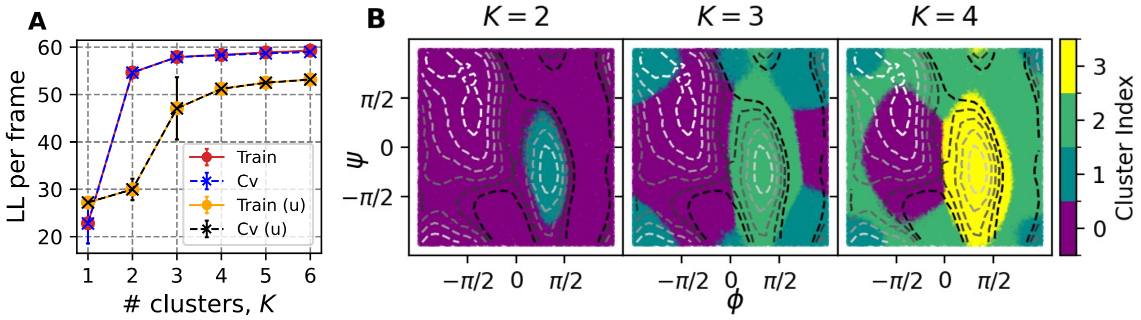

Figure S2: Untemepered MetaD simulation of ADP. (A) Cluster scans obtained with 50K frames, 4 training sets and 10 attempts using rbias frame weights or with uniform weights (labeled 'u'). Training $\ln(L)$ curve is substantially higher with rbias weights, and matches CV curve. (B) Clusterings performed for $K = 2 - 4$ shown by coloring each of 100K sampled points by their cluster assignment. Contour lines indicate the underlying free energy surface as computed from the MetaD simulation via reweighting with rbias frame weights. Contours indicate free energy levels above the minimum from 1 to 11 kcal/mol with a spacing of 2 kcal/mol.

S3 ADP FES computed by evaluating GMM on WT-MetaD samples

In Fig. S3 we assess an alternative approach to estimate an unbiased FES from a GMM object. In this case, we presume that the WT-MetaD simulation produced physically reasonable configurations spanning the configurational landscape of the molecule of interest. To estimate the FES for ADP, we compute a weighted histogram of (ϕ and ψ) where we give as weights the probability of each frame predicted by the GMM, $P(\boldsymbol{x}_i)$ given by Eq. 2. In practice, $P(\boldsymbol{x}_i)$ is computed from exponentiating the log-likelihood of frames within the GMM . We normalize the resulting histogram by samples in each bin, which accounts for the fact that frames were not generated uniformly by WT-MetaD, resulting in a new distribution $\tilde{P}(\phi, \psi)$. The FES is then computed as $F(\phi, \psi) = -k_B \ln \tilde{P}(\phi, \psi)$.

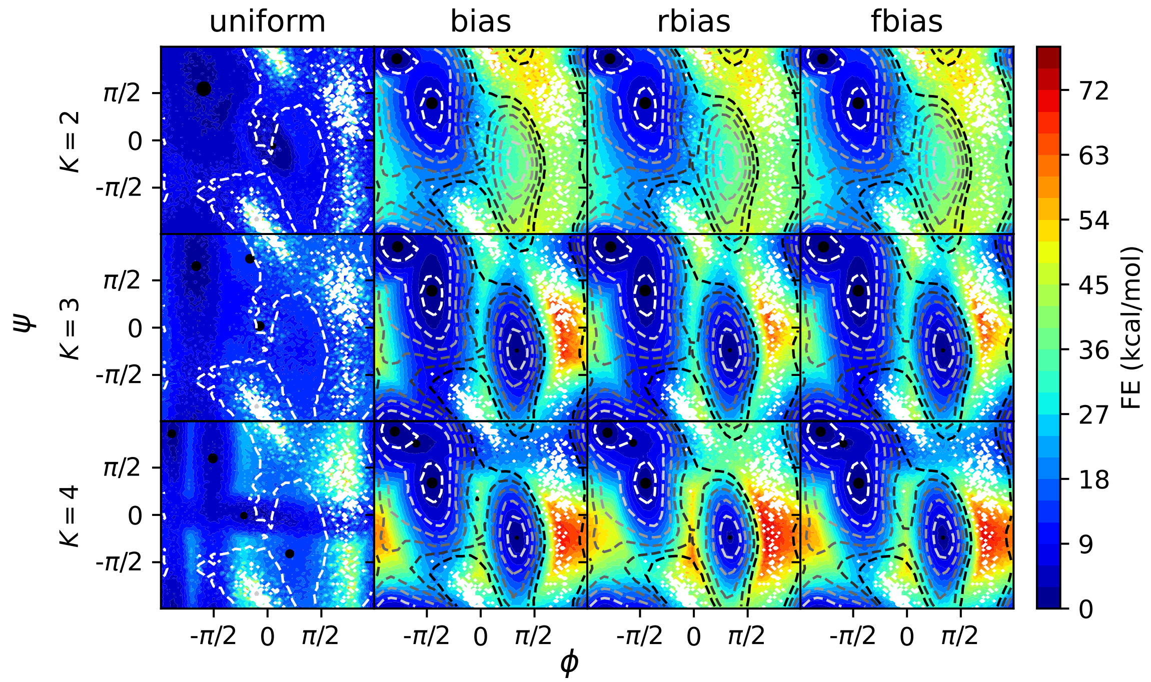

Figure S3: FE profiles obtained from GMM objects trained on BF=10 WT-MetaD data using Monte Carlo procedure. Each column corresponds to a different choice of bias and each row corresponds to a different number of clusters (K) used. Black circles placed on the FEs are the centers calculated from the reference structures corresponding to different clusters, with the size indicating their relative population. Contour lines indicate the underlying free energy surface as computed from the WT-MetaD simulation, positioned at 1.0 to 11.0 kcal/mol with a spacing of 2 kcal/mol above the global minimum.

S4 Error analysis for GMM Free energies

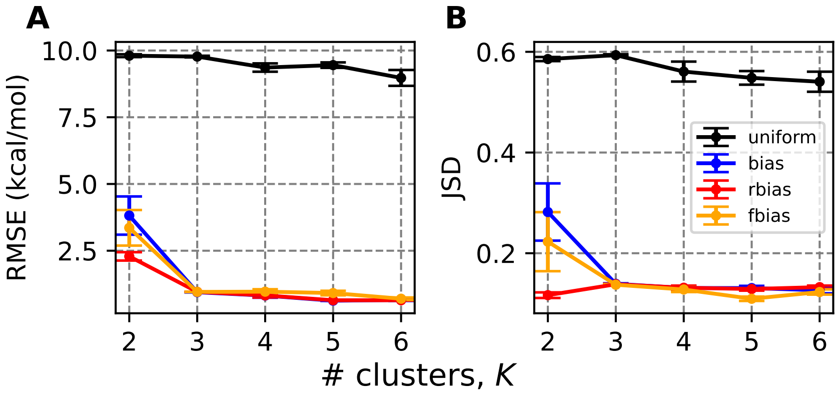

Figure S4: (A) Root mean-squared error for the free energy of ADP GMMs computed for different number of clusters and four different weighting schemes. Error bars are computed from five independent simulations which are fit to separate GMM objects, which are then used to compute free energy surfaces. The reference free energy surface is that computed by summing the Gaussian hills from the WT-MetaD simulation. (B) Same as A, except the Jenson-Shannon distance is computed between the distributions corresponding to $P(\phi, \psi) \propto \exp(-F(\phi, \psi)/(k_B T))$, where $F(\phi, \psi)$ corresponds to either the reference free energy or that computed from the GMM objects.

S5 FEs from GMM for cluster size 5 and 6

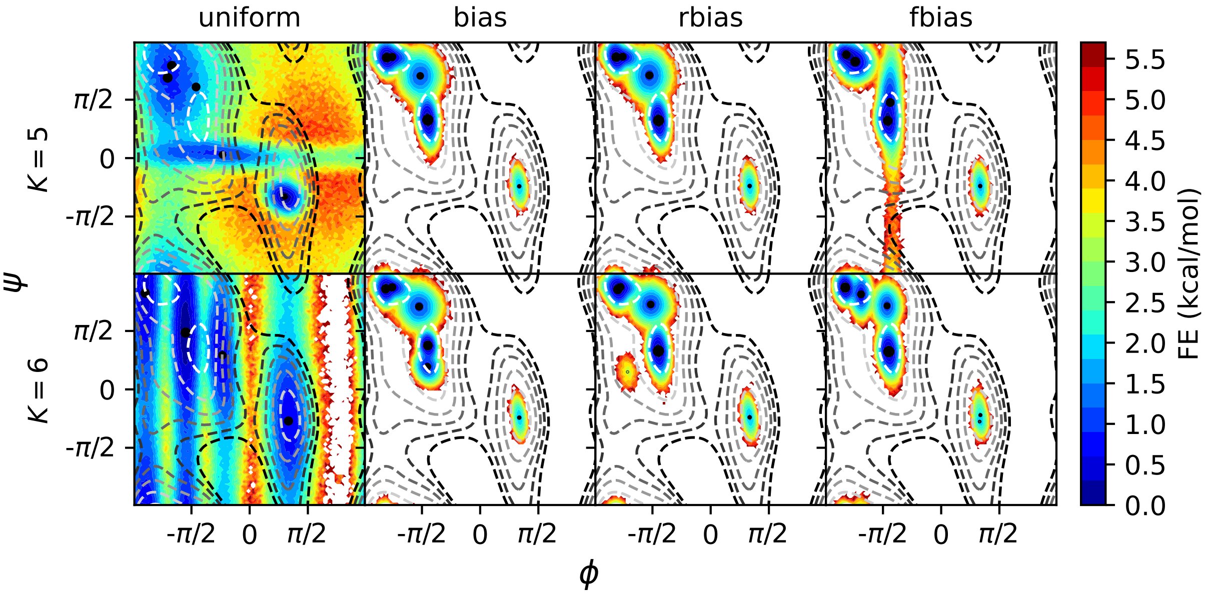

Figure S5: FE profiles obtained from GMM objects trained on BF=10 WT-MetaD data. Each column corresponds to a different choice of bias and each row corresponds to a different number of clusters used. These are computed as unweighted histograms from 1M samples obtained from each GMM object. Black circles placed on the FEs are the centers calculated from the reference structures corresponding to different clusters, with the size indicating their relative population. Contour lines indicate the underlying free energy surface as computed from the WT-MetaD simulation, positioned at 1.0 to 11.0 kcal/mol with a spacing of 2 kcal/mol above the global minimum.

S6 OPES-MetaD simulation of Actin (~ 1us)

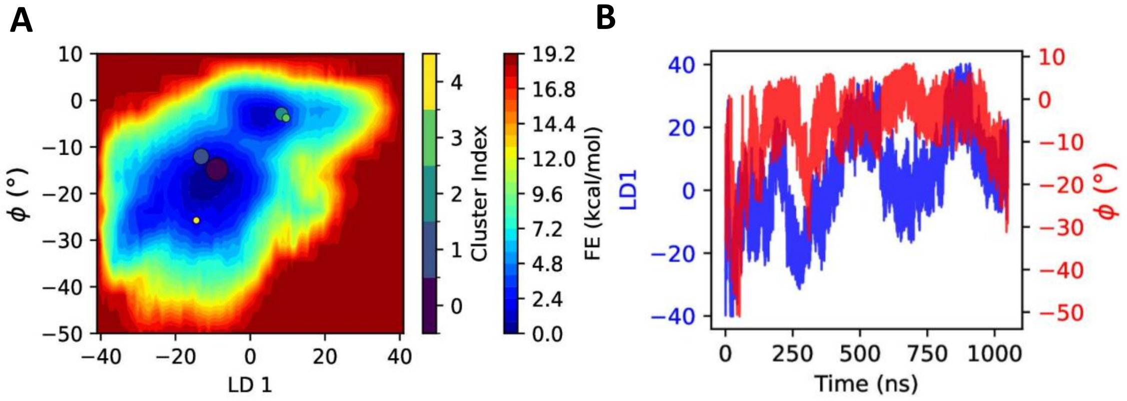

Figure S6: A. The 2D FES obtained from ~1us OPES-MetaD simulation. Colored circles are the locations for cluster centers weighted according to their relative population. B. Time series of LD1 and Dihedral CVs from the same data.

S7 Cluster Scans

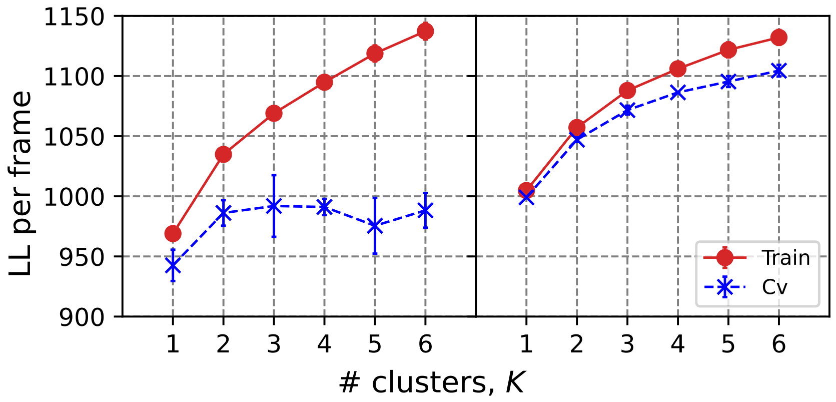

Figure S7: Log likelihood as a function of number of clusters K for the original $\sim 1\mu$s OPES-MetaD trajectory (~21K frames), and using a new set of frames generated by restarting as described in Sec. A1 (~ 153K frames).

S8 Variance of D-loop

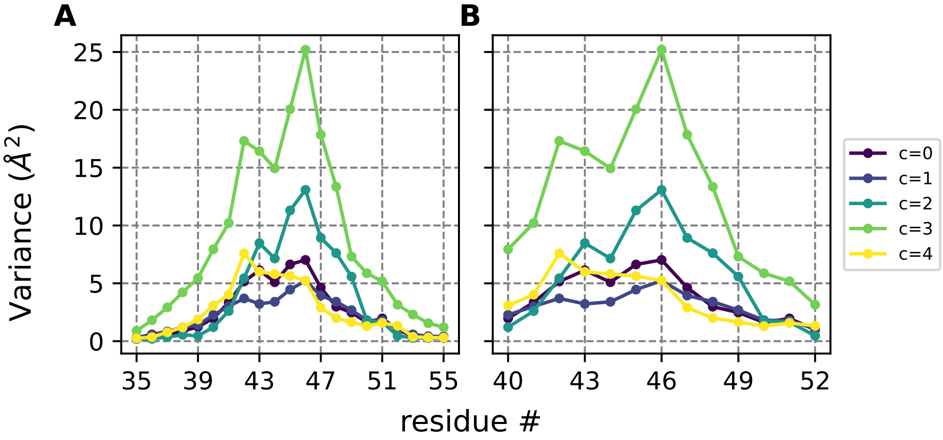

Figure S8: (A) RMSF for residues 35 to 55 within Actin's subdomain 2 including the D-loop. These are extracted from the diagonal of Σ_N for each of five clusters shown in Fig. 4. (B) The same quantity for residues 40 to 52 which represent the core of the D-loop.

S9 Configurational Entropies from GMMs

Table S1: Difference in two configurational entropies computed from probability distributions in dihedral space, comparing all shapeGMM objects with metadynamics taken as ground truth (GT). $\Delta S^{K,C}_{\text{config}} = S^{K,C}_{\text{config}} - S^{GT}_{\text{config}}$, where K= # Clusters, C= choice of weight. To compute $S^{K,C}_{\text{config}}$, 1M samples are generated from the shapeGMM object and a 2D normalized probability distribution is calculated in dihedral space with generated data. All S_{config} values are calculated using Eq.10. uniform$_{modf}$ represents uniform weight shapeGMM objects where the cluster populations are reweighted using final bias weights after the shapeGMM fit. To reweigh, we update the weights for each cluster in a given shapeGMM object with the sum of normalized fbias weights for all frames assigned to that cluster in the uniform scheme. ΔS_{config} is always less for the weighted objects compared to uniform weights irrespective of cluster sizes.

$\Delta S_{\text{config}}/k$					
# Clusters, K	choice of weight, C				
	uniform	bias	rbias	fbias	uniform$_{modf}$
2	2.42	-0.34	-0.31	-0.34	2.42
3	2.18	-0.80	-0.79	-0.80	2.18
4	1.48	-0.82	-0.82	-0.82	1.48
5	2.23	-0.80	-0.78	-0.69	2.24
6	1.52	-0.74	-0.83	-0.80	1.52
